# Effects of Europium Complex on Thermal and Photoluminescence Properties of Polyurethane-Europium Materials

**DOI:** 10.3390/polym15051064

**Published:** 2023-02-21

**Authors:** Lijun Gao, Liuyang Li, Yunqiu Li, Congcong He, Liming Zhou, Xiongwei Qu, Shaoming Fang

**Affiliations:** 1 School of Chemical Engineering and Technology, Hebei University of Technology, No.8 Guangrong Road, Tianjin 300130, China; 2 Henan Provincial Key Laboratory of Surface & Interface Science, School of Material and Chemical Engineering, Zhengzhou University of Light Industry, Zhengzhou 450002, China

**Keywords:** europium complex, polyurethane, composite, transparency, photoluminescence

## Abstract

A europium complex with double bonds was synthesized with crotonic acid as the ligand and a europium ion as the center ion. Then, the obtained europium complex was added to synthesized poly(urethane-acrylate) macromonomers to prepare the bonded polyurethane-europium materials by the polymerization of the double bonds in the complex and the poly(urethane-acrylate) macromonomers. The prepared polyurethane-europium materials had high transparency, good thermal stability and good fluorescence. The storage moduli of polyurethane-europium materials are obviously higher than those of pure polyurethane. Polyurethane-europium materials exhibit bright red light with good monochromaticity. The light transmittance of the material decreases slightly with increases in the europium complex content, but the luminescence intensity gradually increases. In particular, polyurethane-europium materials possess a long luminescence lifetime, which has potential applications for optical display instruments.

## 1. Introduction

Rare earth complexes are widely used in the field of molecular probes and molecular tracking, and so on, due to their strong photosensitizing effects [1,2,3,4], long fluorescence lifetime [5,6,7,8,9] and good luminescent performance [10,11,12,13,14,15,16,17]. However, their application has been greatly limited for macromolecule complexes due to the poor compatibility, solubility and processability of these complexes. Polymer composites have emerged to make up for these defects by introducing rare earth complexes into polymers as a dopant to take advantage of the polymers properties—in particular, their easy processing performance.

Doping and bonding methods are usually used to prepare polymer–rare earth composite materials. The doping method is easy to operate and implement, but it is prone to cause the rare earth complex to disperse unevenly and separate from the polymer [18,19,20,21,22,23,24,25,26,27,28,29], resulting in the reduction of prepared composite’s properties. Ethylene-methyl acrylate copolymer (EMA) films doped with Eu(Ⅲ) organic complexes are used to obtain Eu-EMA films with excellent fluorescence performance, but a certain amount of the Eu(Ⅲ) organic complex particles separate out, and the particles attach and aggregate to the surface of the film [30]. In order to enlarge its scope of application and to reduce agglomeration, the other type of polymer–rare earth composite is usually prepared by the bonding method. This method is used to bond the rare earth complex to the polymer molecular chain through a reaction or coordination method, so that the rare earth complex is not easy to separate from the polymer matrix and can be evenly dispersed in the matrix [31,32,33,34,35,36,37,38]. A type of mechanochemiluminescent polymer with red light emission can be accessed by incorporating the (Eu(TTA)_3_(Aphen)) complex into the side chain of a 1,2-dioxetane cross-linked poly(methyl acrylate). The sensitive, stretchable device with a polymer film as an emissive layer can emit patterned red light upon deformation. This sensitized mechanoluminescence design thus expands the useful area of luminescent stress probes [39]. However, due to the difficulties in the synthesis of rare earth complexes with active functional groups and the coordination between rare earth materials and polymers, the bonding method is not easy to implement. When polymer–rare earth composites are used as transparent optical devices, the composites are required to have high light transmittance. However, the addition of rare earth compounds into polymers often reduces the light transmittance of the matrix materials. Polystyrene (PS) and poly (methylmethacrylate; PMMA)-grafted Er–Yb co-doped transparent ternary nanocomposites have good fluorescence performance, but the rare earth compounds reduce the light transmittance of the material. When the thickness of the composite is greater than 4 mm, the light transmittance is below 55% [40]. Alkaline earth aluminate (AEA) can also enhance the photochromic and long-lasting photoluminescent performances of PMMA plastic. However, the light transmittance of PMMA sheets decreases largely with increasing levels of AEA [41]. Polyurethanes are a class of polymers with adjustable structures and properties that can vary from rubbery materials to glassy thermoplastics and from linear to thermosetting materials [42]. Polyurethanes can be used in extensive applications including optics, automotive parts, and optoelectronics due to their outstanding properties [43,44,45,46,47,48], such as their transparence in a wide wavelength range, their chemical stability, their good dimensional stability, etc. The introduction of rare earth complexes into polyurethanes by the bonding method can not only solve the problem that rare earth complexes are not easy to process, but can also give polyurethanes better optical properties.

In this paper, a europium complex with active vinyl groups was synthesized with europium oxide and crotonic acid. Then, the obtained europium complex was introduced into polyurethane to prepare polyurethane-europium materials by the polymerization of poly(urethane-acrylate) macromonomers and europium complexes. Bonding rare earth complexes to the polyurethane molecular chain avoids the dissociation and precipitation of the complex, which can ensure the stable luminescence performance of the material. At the same time, europium complexes have good compatibility with polyurethane, which can make the polyurethane–europium material have high transmittance. The influences of the europium complex content on the properties of polyurethane–europium materials are studied systemically here.

## 2. Materials and Methods

### 2.1. Materials

Lactic acid was purchased from the Tianjin Kaitong Chemical Co., Ltd. Polyethylene glycol 600 (PEG 600) was provided from the Tianjin Kermel Chemical Co., Ltd. Isophorone diisocyanate (IPDI) was supplied by the Shanghai NCM Hersbit Chemical Co., Ltd. Europium oxide (Eu_2_O_3_) was purchased from Shanghai Yue-Long Non-Ferrous Metals. Crotonic acid was supplied by Aladdin Reagent Co., Ltd., Shanghai China and 2-hydroxyethyl methacrylate (HEMA) was purchased from the Tianjin Chemical Reagent Research Institute. Dibutyltin dilaurate (DBTL) was provided by the Shanghai Far Navigation Chemical Reagent Factory and 2,2′-Azobis(2-methylpropionitrile; AIBN) was purchased from the Tianjin Fuchen Chemical Reagants Factory, recrystallized from alcohol and dried by vacuum distillation. Other materials were standard laboratory reagents and were used as received.

### 2.2. Synthesis of the Europium Complex

A mixture of 5 mmol Eu_2_O_3_ and 30 mmol crotonic acid was dissolved in 60 mL distilled water, stirred and heated under reflux at 90 °C for 2.5 h. After the mixture solution was filtered to remove unreacted Eu_2_O_3_, the obtained filtrate was heated to remove distilled water by reduced pressure distillation. The obtained powder was carefully washed with ethyl ether and absolute alcohol at least 5 times, followed by drying in a vacuum desiccator for 24 h. Finally, the europium complex was obtained and stored in a dryer. The europium complex needed to be dried again before each use to remove any moisture in the complex.

### 2.3. Preparation of Polyurethane–Europium Materials

IPDI:PEG 600:actic acid:HEMA was weighed at a molar ratio of 4:1:1:4, and DBTL (as a catalyst) and AIBN (as an initiator) were added at 0.3 wt% of the total weight. The Europium complex was added at 0 wt%, 0.5 wt%, 1 wt%, 2 wt%, 2.5 wt% and 3 wt% of the total weight. The total weight refers to the sum of the weights of the IPDI, PEG-600, lactic acid and HEMA.

The appropriate amounts of IPDI and lactic acid were placed in a 250 mL round-bottomed flask. After mixing evenly at 25 °C, DBTL was added and stirred for 5 min. Then, PEG 600 was added to the reaction system and the reaction was continued for 40 min to obtain the intermediate. Then, a predetermined amount of HEMA was added to the above intermediate and stirred for 40 min to obtain poly(urethane-acrylate) macromonomers with double bonds. After that, the synthesized europium complex and AIBN were introduced into the macromonomers to obtain the mixture. Then, the mixture was poured into a preheated glass mold at room temperature and reacted at 30 °C, 40 °C, 50 °C and 60 °C for 1 h, respectively, and then heated to 70 °C for an additional 24 h. Finally, polyurethane–europium materials with a certain thickness (5 mm) were obtained during the thermosetting molding process. Different polyurethane–europium materials can be obtained by changing the content of europium complex.

### 2.4. Characterization

Fourier transform infrared (FTIR) spectra of samples were recorded on a TEMSOR27 FTIR spectrometer, and scanning times were 16 times in the wave number range of 500–4000 cm^−1^. The infrared spectrum of the europium complex was obtained by the KBr method, and infrared spectra of the polyurethane–europium materials were tested with plates of 5 mm thickness. UV-vis spectra were carried out using a Hitachi U-3900H UV-vis spectrophotometer. The wavelength range was 200–600 nm. The scanning speed was 30 nm/min and the slit width was 2 nm. WAXD measurements were made using a Bruker D8 ADVANCE X-ray diffractometer. The angular 2 theta diffraction range was between 5° and 70°. The data were collected with an angular step of 0.04° at 0.5 s per step. Cu Kα radiation was obtained from a copper X-ray tube operated at 40 kV and 30 mA. The weight loss of the samples under a N_2_ atmosphere (100 mL/min) was measured using a Perkin–Elmer Diamond TG/DTA thermogravimeter and the samples, with weight of about 5 mg, were heated from room temperature to 700 °C at a heating rate of 10 °C/min in an alumina crucible. The initial thermal decomposition temperature is defined as the temperature corresponding to the intersection of the tangent of the point with the fastest thermal decomposition rate on the TG curve and the initial baseline extension line. The internal morphologies of the samples were observed using a transmission electron microscope (TEM, JEOL, JEM-2100). Polyurethane–europium materials, with different mass contents of europium complex, were cut into thin slices by an ultrathin slicer for testing. Their dynamic thermal mechanical properties were determined using Q800 dynamic mechanical analysis (DMA) with a rate of 3 °C/min in a temperature range from 30 °C to 200 °C. Samples with a size of 35.5 mm × 12.5 mm × 5 mm were mounted in a single cantilever. Excitation and emission spectra and fluorescence lifetimes were obtained by a time-resolved and steady-state fluorescence spectrometer (FLS 980, Edinburgh Instruments, Livingston, UK) with an excitation slit of 5 nm width at room temperature. The europium complex was tested with powder. The Polyurethane–europium materials were tested with 5 mm thick plates. Excitation spectra and emission spectra were measured at an emission wavelength of 617 nm and excitation wavelength of 394 nm. The luminescence lifetime was estimated from the fluorescence decay curves by software followed by one exponential and bi-exponential decay kinetics. Transparencies were measured using a WGT-S Ubest-35 spectrometer with a white halogen light. The dimensions of the samples were 50 mm × 50 mm × 5 mm.

## 3. Results and Discussion

### 3.1. Structure of Polyurethane–Europium Materials

**Figure 1:**
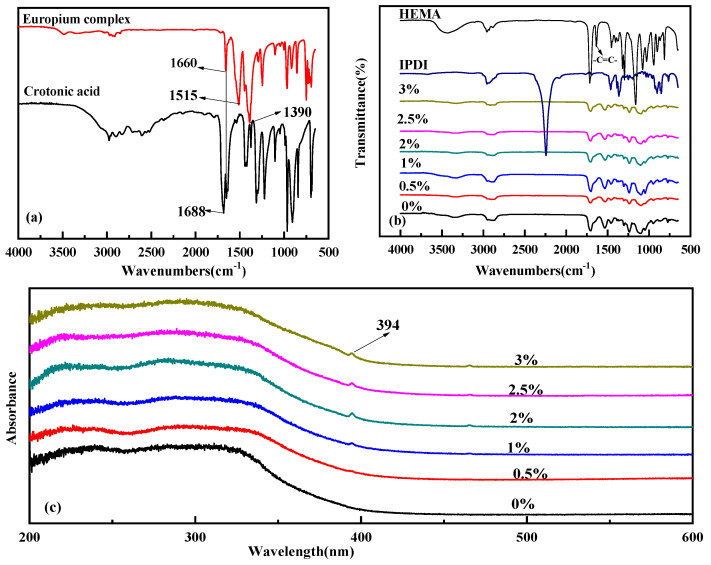
FTIR (**a**,**b**) and UV (**c**) spectra of the europium complex and polyurethane−europium materials with different mass contents of europium complex.

It can be seen from Figure 1a that the characteristic peak of a carboxyl group appears at 1688 cm^−1^ in crotonic acid. However, for the europium complex, the 1688 cm^−1^ peak disappeared and two strong characteristic peaks appeared at 1390 cm^−1^ and 1515 cm^−1^, corresponding to the symmetric(υ_s_ (COO-)) and antisymmetric stretching vibration absorption (υ_as_ (COO-)) of carboxyl groups; Δ*ν = ν*_as_ (-COO^−^) − *ν*_s_ (-COO^−^) = 125 cm^−1^, which is less than 200 cm^−1^ [49], indicating that crotonic acid participated in the coordination reaction to form the europium complex. The europium complex still had a double bond characteristic peak at 1660 cm^−1^, implying that the europium complex contained an active group (double bond).

The FTIR spectra of the polyurethane–europium materials are shown in Figure 1b. Polyurethane–europium materials have the same absorption peaks. The FTIR spectra of the polyurethane–europium materials show that a urethane structure was demonstrated by the absorption bands at around 3350 cm^−1^ (N-H stretching), 1530 cm^−1^ (C-N stretching, combined with N-H out-of-plane bending), 1100 cm^−1^ (-O- asymmetrical stretching) and 1700 cm^−1^ (C=O stretching). The absorption peak near 2240 cm^−1^ disappeared, indicating that the isocyanate group (–NCO) of IPDI reacted completely. Moreover, the absorption peak at 1630–1660 cm^−1^ (-C=C-) disappeared, implying that the double bonds of the HEMA and europium complex reacted completely. The results confirm that the structure of the polyurethane–europium materials fully accorded with those of the design.

The UV spectra of the polyurethane–europium materials are shown in Figure 1c. The electron transitions of π-π * and n-π * occurred at around 220 nm and 310 nm, respectively, so a wide absorption band was present at 200–350 nm. When the content of the europium complex reached 1 wt%, a small absorption peak appeared at 394 nm, which was due to the electron transition from the ^7^F_0_ to the ^5^L_6_ of the Eu^3+^. Furthermore, as the europium complex content increased, the absorption peak at 394 nm became gradually obvious. As the UV-visible absorption peaks of the polyurethane–europium materials were below 400 nm, the materials had high transmittance for light greater than 400 nm.

### 3.2. Dispersion of Europium Complex in Polyurethane

**Figure 2:**
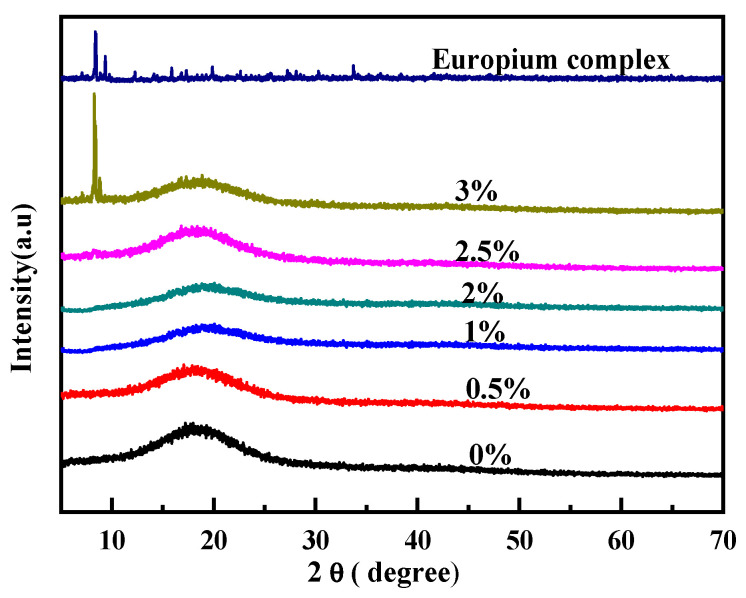
XRD patterns of the europium complex and polyurethane–europium materials with different mass contents of europium complex.

Figure 2 shows the XRD patterns of the europium complex and polyurethane–europium materials. When the mass content of the europium complex was less than 2.5 wt%, the diffraction peaks of the pure polyurethane and polyurethane–europium materials were broad and there was no obvious crystal peak. As such, the materials exhibited an amorphous state. However, when the mass content of the europium complex was increased to 3 wt%, some narrow diffraction peaks centered at about 8.4° occurred. When compared with the diffraction peaks of the europium complex, these peaks coincided with the diffraction peaks of the europium complex. This phenomenon was due to the uneven dispersion of the europium complex in the polyurethane matrix, so that the europium complex had a slight reunion phenomenon when its content increased.

**Figure 3:**
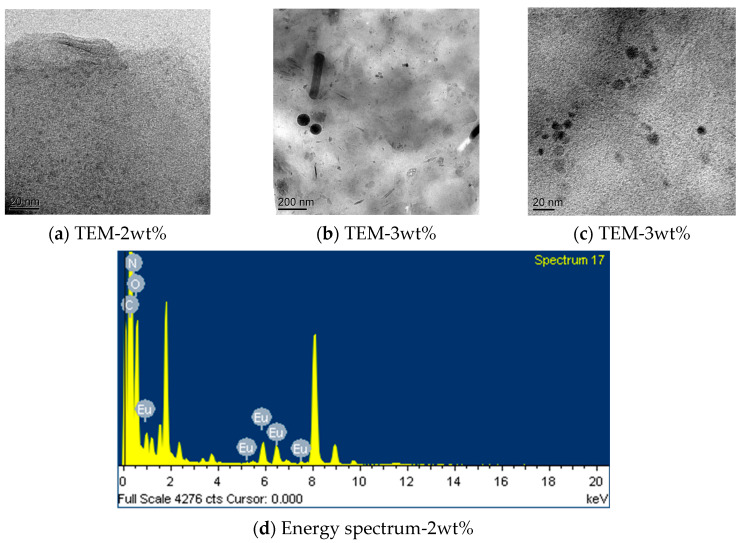
TEM images (**a**–**c**) and energy spectrum (**d**) of the polyurethane–europium materials containing 2 wt% and 3 wt% europium complex.

Figure 3 shows the TEM images and energy spectrum of the polyurethane–europium materials containing 2 wt% and 3 wt% europium complex. When the content of the europium complex was 2 wt%, the complex was dispersed evenly in the polyurethane matrix (Figure 3a). At the same time, it can be seen that the polyurethane–europium material contained the Eu element in the energy spectrum of the polyurethane–europium material (Figure 3d). However, when the content of the complex was up to 3 wt%, there was a small amount of agglomeration phenomena in the polyurethane matrix (Figure 3b,c).

### 3.3. Transparency of Polyurethane–Europium Materials

**Figure 4:**
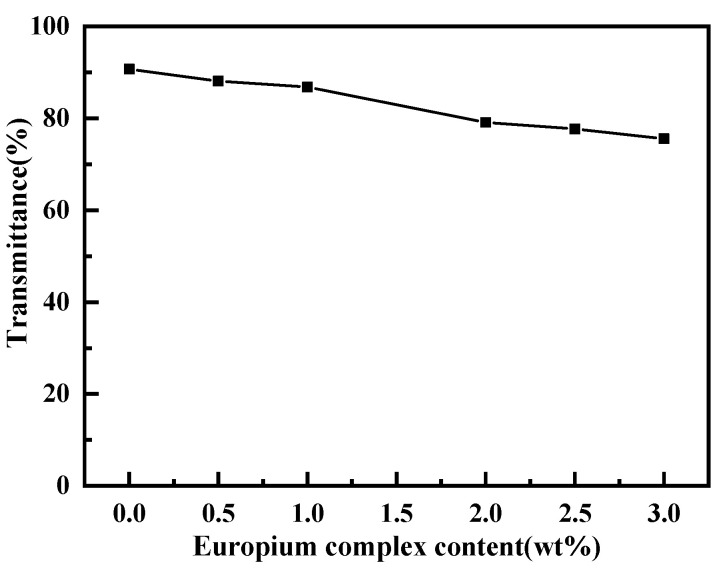
Light transmittance of the polyurethane–europium materials with different mass contents of europium complex.

The light transmittance of the polyurethane–europium plates with a thickness of 5 mm is shown in Figure 4. The pure polyurethane material had the highest transmittance of 90.7%. When the content of the europium complex was 0.5 wt%, 1 wt%, 2 wt%, 2.5 wt%, and 3 wt%, the transmittance of the polyurethane–europium material was 88.1%, 86.8%, 79.1%, 77.7% and 75.6%, respectively. It can be seen that the higher the content of europium complex, the lower the transparency of the material. This may be due to the poor compatibility of the complex with the polyurethane or the uneven dispersion of the complex in the polyurethane, which caused the complex to agglomerate locally and form tiny particles (Figure 3b). These tiny particles can change the direction of light, cause light scattering loss and reduce transmittance. However, the light transmittance of all the polyurethane–europium plates was above 75%, so the materials were still highly transparent and could be used as optical materials.

### 3.4. Thermal Stability of Polyurethane–Europium Materials

**Figure 5:**
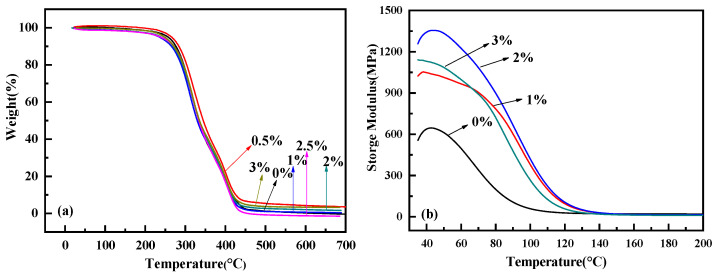
TG curves (**a**) and storage moduli UV (**b**) of the polyurethane–europium materials with different mass contents of europium complex.

The TG curves of the polyurethane–europium materials with different mass contents of europium complex are shown in Figure 5a. The thermal degradation of the polyurethane–europium materials was divided into two steps: The first was that the urethane group on the polyurethane main chain was broken at the C-O bond at 240–340 °C; it decomposed into isocyanates and polyols, and then further decomposed into amines, olefins and CO_2_. Another loss process occurred at 340–450 °C, accompanied by the formation of a carbonaceous residue and europium compound. The initial thermal decomposition temperature of the polyurethane–europium materials was above 230 °C, which indicates that the materials had good thermal stability. Besides this, as the content of europium complex increased, the thermal stability of the material did not change significantly—indicating that the addition of the europium complex did not reduce the thermal stability of the PU material.

Figure 5b shows the storage moduli of the polyurethane–europium materials with different mass contents of europium complex. The storage modulus, also known as the elastic modulus, refers to the amount of stored energy due to elastic (reversible) deformation when the material deforms; it reflects the elasticity of the material. It can be seen from Figure 5b that the storage modulus of pure polyurethane decreased with increases in temperature and gradually stabilized at about 120 °C. The storage moduli of all the polyurethane–europium materials were obviously higher than those of pure polyurethane at the same temperature, indicating that the ability of the polyurethane–europium materials to store elastic deformation energy increased. When the content of the complex was less than 2 wt%, the storage modulus of the polyurethane–europium material gradually increased with increases in the content of the complex. This is mainly because the copolymerization of the europium complex and poly(urethane-acrylate) macromonomers increased the crosslinking degree and rigidity of materials, which made the material less easy to deform, resulting in an increase in the elastic modulus. However, the storage modulus decreased when the content of the complex reached 3 wt%; this was mainly due to the partial agglomeration of the complex in the polyurethane matrix. This phenomenon is consistent with results of XRD and TEM. The temperature at the inflection point of the storage modulus curve is usually taken as the glass transition temperature. It can be seen that the rare earth complex significantly increased the glass transition temperature of the polyurethane–europium material. These results show that the addition of the europium complex can improve the storage modulus and work temperature, and can widen the application range of polyurethane.

### 3.5. Luminescence of Polyurethane–Europium Materials

**Figure 6:**
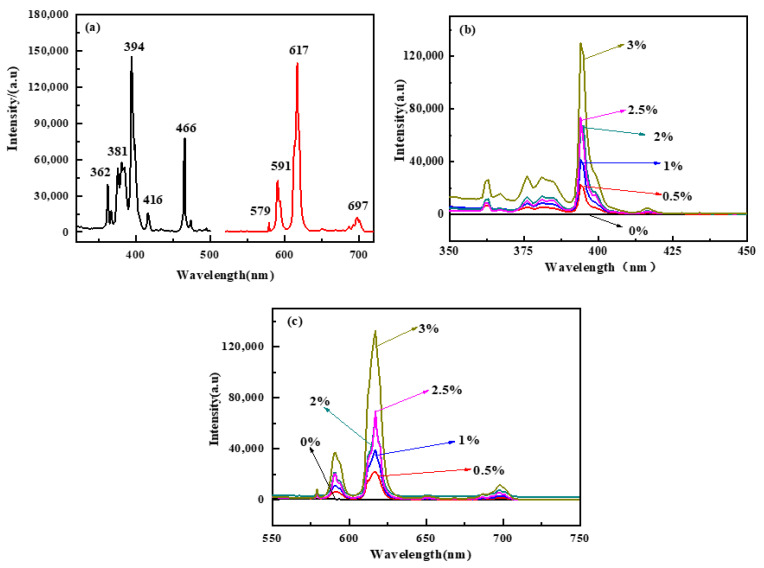
Excitation spectra with an emission wavelength of 617 nm and emission spectra at an excitation wavelength of 394 nm of the europium complex powders (**a**) and polyurethane–europium materials with different mass contents of europium complex (**b**,**c**) at room temperature.

Figure 6 demonstrates the excitation and emission spectra of the europium complex and polyurethane–europium materials with different mass contents of europium complex. The excitation spectra of the europium complex and polyurethane–europium materials were investigated at an emission wavelength of 617 nm. In Figure 6a, the peaks at 362 nm, 381 nm, 394 nm, 416 nm and 465 nm were assigned to ^7^F_0_→^5^D_4_, ^7^F_0_→^5^G_2_, ^7^F_0_→^5^L_6_, ^7^F_0_→^5^D_3_, ^7^F_0_→^5^D_2_ transitions of the Eu^3+^ ion [50]. The excitation spectra of the polyurethane–europium materials were similar to those of the europium complex. Moreover, with increases in the content of the europium complex, the excitation spectrum of the material was gradually enhanced. The emission spectra were investigated by fixing a excitation wavelength of 394 nm, and the results are shown in Figure 6c. The emission spectra of the polyurethane–europium materials were similar to those of the europium complex (Figure 6a). The characteristic emission peaks at 591 nm and 617 nm corresponded to the ^5^D_0_→^7^F_1_ (magnetic dipole transition) and ^5^D_0_→^7^F_2_ (electric dipole transition) transitions of the Eu^3+^ ion, respectively [51]. The luminescence intensity of the complex at 617 nm was distinctly stronger than that of the other emission wavelength, and exhibited bright red light with good monochromaticity [52]. The luminescence properties of the complex were influenced by the interaction between the ligand and central ion. The emission peak of the europium complex was narrow and high, which indicated that the ligand sensitized the characteristic emission of Eu^3+^ ion. The ligand absorbed energy and effectively transferred it to the central Eu^3+^ ion, so the europium complex exhibited strong luminescence intensity. 

Moreover, it can be also observed that the luminescence intensity of the polyurethane–europium increased gradually with increasing amounts of the europium complex and with no fluorescence concentration quenching within the range of the europium complex contents from 0.5 wt% to 3 wt% (Figure 6c). This is due to the fact that the europium complex had been bonded onto the polyurethane to prevent its agglomeration to a certain extent, endowing the materials with more stable properties. The results indicate that the polymerization of the europium complex and poly(urethane-acrylate) macromonomers had altered the local environment of the Eu^3+^, which lowered the degree of symmetry of the local environment.

**Figure 7:**
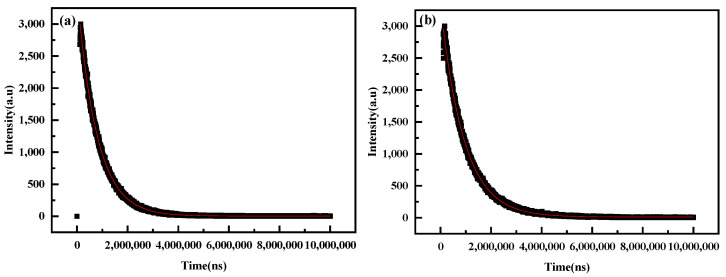
The fluorescence decay curves of the europium complex powders (**a**) and polyurethane–europium material containing 3 wt% europium complex (**b**) at 617 nm (λex = 394 nm) at room temperature.

Figure 7a shows the excited state decay curve of the emission of the europium complex at 617 nm, under a 394 nm excitation wavelength at room temperature. The decay curve was fitted by a mono-exponential function(y = A_1_ × exp(−x/t_1_) + y_0_); the Luminescence lifetime equation was I(t) = 3482.586exp(−t/779809.482) + 2.761 and the luminescence lifetime was calculated to be 0.780 ms. Meanwhile, the luminescence decay curve (Figure 7b) of the polyurethane–europium containing 3 wt% europium complex was well-fitted by a bi-exponential function(y = A_1_ × exp(−x/t_1_) + A_2_ × exp(−x/t_2_) + y_0_), and the obtained fitting curve equation was I(t) = 1660.884exp(−t/920974.651) + 1660.884exp(−t/920967.633) + 10.696. The fluorescence lifetime was calculated by t = (A_1_ × t_1_^2^ + A_2_ × t_2_^2^)/(A_1_ × t_1_ + A_2_ × t_2_), and the obtained fluorescence lifetime of the polyurethane–europium material was 0.921 ms.

The fitting curves and fluorescence life calculation methods of the other polyurethane–europium materials containing 0.5 wt%, 1 wt%, 2 wt% or 2.5 wt% europium complex were the same as those of the polyurethane–europium materials containing 3 wt% europium complex. Their fitting fluorescence decay curves and calculated fluorescence life are shown in Table 1. The addition of the europium complex to polyurethane did not reduce the fluorescence lifetime of the complex, and the fluorescence lifetime of the polyurethane–europium materials was slightly longer than that of the pure europium complex (0.780 ms). This may be because the complex bonds to the PU to make the europium complex disperse uniformly in the PU matrix. At the same time, the crosslinked grid of the thermosetting polyurethane prevents the aggregation of the europium complex to some extent.

## 4. Conclusions

The obtained polyurethane–europium materials produced by introducing the europium complex into polyurethane had good luminescent properties. The polyurethane–europium materials exhibited an amorphous state and the absorption peak of their UV-Vis spectra was below 400 nm, resulting in their high transmittance (more than 75%) under white light. When the content of europium complex was less than 3 wt%, the distribution of the europium complex in the polyurethane matrix was basically uniform. The thermal decomposition temperature of the polyurethane–europium materials was above 230 °C, indicating that the materials had good thermal stability. The content of the complex had a great influence on the storage modulus and photoluminescence performances of the materials. The addition of the europium complex significantly increased the storage modulus and glass transition temperature of the material. When the content of the europium complex was 2 wt%, the storage modulus of the material increased by more than one time compared with the pure PU, and the glass transition temperature was increased by nearly 30 °C. The fluorescence intensity of the polyurethane–europium materials regularly increased with increases in the europium complex. As the content of europium complex had the opposite effect on the transmittance and fluorescence intensity of the material, the amount of europium complex should be comprehensively considered when it is used as a transparent optical material. The introduction of the complex into the polyurethane did not reduce its fluorescence lifetime, and the fluorescence lifetimes of all the polyurethane–europium materials were slightly longer than that of the europium complex. The polyurethane–europium materials not only had high transparency and showed easy molding of the polyurethane materials, but also had the excellent luminescent properties of the europium complex, with potential applications as photoluminescent materials—especially in fields with high transmittance requirements.

## Figures and Tables

**Table 1:** Fluorescence decay curve and lifetime of polyurethane–europium materials with different mass contents of europium complex.

Content of Eu Complexes (wt%)	Fluorescence Decay Curve	Fluorescence Lifetime (ms)	R^2^
0.5	I(t) = 351.260exp(−t/429562.543) + 940.652exp(−t/1164870) + 2.136	1.075	0.997
1	I(t) = 1384.104exp(−t/769872.31) + 371.719exp(−t/1448860)+ 0.625	0.997	0.998
2	I(t) = 1722.242exp(−t/818073.406) + 1722.24263exp(−t/818072.250) + 4.340	0.818	0.998
2.5	I(t) = 1758.733exp(−t/784677.206) + 1758.733exp(−t/784676.809) + 3.644	0.786	0.998
3	I(t) = 1660.884exp(−t/920974.651) + 1660.884exp(−t/920967.633) + 10.696	0.921	0.998

## Data Availability

Not applicable.

## References

[1] Dasari S., Patra A.K. (2015). Luminescent europium and terbium complexes of dipyridoquinoxaline and dipyridophenazine ligands as photosensitizing antennae: Structures and biological perspectives. Dalton Trans..

[2] Baek N.S., Kim Y.H., Lee D.H., Seo K.D., Kim H.K. (2009). Effect of coordination environment on the photophysical properties of luminescent europium(III) complexes. Bull. Korean Chem. Soc..

[3] Ung P., Clerc M., Huang H., Qiu K., Chao H., Seitz M., Boyd B., Graham B., Gasser G. (2017). Extending the excitation wavelength of potential photosensitizers via appendage of a kinetically stable terbium(III) macrocyclic complex for applications in photodynamic therapy. Inorg. Chem..

[4] Manseki K., Hasegawa Y., Wada Y., Yanagida S. (2005). Photosensitized luminescence of thermostable polynuclear Eu(III) complexes. J. Lumin..

[5] Zhu M.M., Zhang Z., Ren N., Wang S.P., Zhang J.J. (2019). Rare earth complexes with 3,4-dimethylbenzoic acid and 2,2:6′,2″-terpyridine: Synthesis, crystal structures, luminescence and thermodynamic properties. Inorg. Chim. Acta.

[6] Chen Y.M., Li L., Zhang Q.C., Liu S.S., Tian Z.F., Ju Z.H. (2020). Effects of calcium ions on crystal structure and luminescence properties of six rare earth metal complexes. J. Solid State Chem..

[7] Zhang R.J., Yang K.Z., Yu A.C., Zhao X.S. (2000). Fluorescence lifetime and energy transfer of rare earth β-diketone complexes in organized molecular films. Thin Solid Films.

[8] Zhang J., Li W.X., Ao B.Y., Feng S.Y., Xin X.D. (2014). Fluorescence enhancement of europium(III) perchlorate by benzoic acid on bis(benzylsulfinyl)methane complex and its binding characteristics with the bovine serum albumin (BSA). Spectrochim. Acta A.

[9] Guo L.W., Li X., Shi X.Y., Sun X.J., Sun X.L. (2009). Enhanced luminescence of rare-earth Tb(III) by Tm(III) in bis(benzoylmethyl) sulfoxide complexes and intra-molecular energy transfer. J. Lumin..

[10] Jiu H.F., Liu G.D., Zhang Z.J., Fu Y.H., Chen J.C., Fan T., Zhang L.X. (2011). Fluorescence enhancement of Tb(III) complex with a new beta-diketone ligand by 1,10-phenanthroline. J. Rare Earths.

[11] Liu C.L., Zhang R.L., Lin C.S., Zhou L.P., Cai L.X., Kong J.T., Yang S.Q., Han K.L., Sun Q.F. (2017). Intraligand charge transfer sensitization on self-assembled europium tetrahedral cage leads to dual-selective luminescent sensing toward anion and cation. J. Am. Chem. Soc..

[12] Wang Z.H., Zhu Q., Wang X.J., Li X.D., Sun X.D., Kim B.N., Li J.G. (2019). Multi-color luminescent m-LaPO_4_:Ce/Tb monospheres of high efficiency via topotactic phase transition and elucidation of energy interaction. Inorg. Chem..

[13] Li Q.Y., Tang P.S., Hou R.P., Zhou Y.F., Chen H.F. (2021). Effect of ligands on luminescent properties of rare earth europium complexes. Integr. Ferroelectr..

[14] Barja B., Aramendia P., Baggio R., Garland M.T., Pena O., Perec M. (2003). Europium(III) and terbium(III) trans-2-butenoates: Syntheses, crystal structures, and properties. Inorg. Chim. Acta.

[15] Sharma G., Narula A.K. (2015). Synthesis of Eu(III) complexes with 2-aminopyridine and 1,10-phenanthroline: Structural, optical, thermal and morphological studies. Sens. Actuators B Chem..

[16] Devi R., Bala M., Khatkar S.P., Taxak V.B., Boora P. (2016). Investigations of luminescent behavior and intramolecular energy transfer mechanism of europium(III) complexes with fluorinated β-ketoester ligand. J. Fluor. Chem..

[17] Singh-Wilmot M.A., Sinclair R.A., Kahwa I.A., Lough A.J. (2017). Eu^3+^ substitutional defects and their effect on the luminescence spectral and decay dynamics of sal-type one dimensional rare earth coordination polymers and trinuclear complexes. J. Lumin..

[18] Zhao X.Y., Song L., Zhao R., Tan M.C. (2019). High-performance and flexible shortwave infrared photodetectors using composites of rare earth-doped nanoparticles. ACS Appl. Mater. Interfaces.

[19] Wang H.N., Fang L., Zhang Z., Epaarachchi J., Li L.Y., Hu X., Lu C.H., Xu Z.Z. (2019). Light-induced rare earth organic complex/shape-memory polymer composites with high strength and luminescence based on hydrogen bonding. Compos. Part A—Appl. Sci. Manuf..

[20] Zhou L., Yang H.W., Zhang Z., Liu Y., Epaarachchi J., Fang Z.G., Fang L., Lu C.H., Xu Z.Z. (2022). A Flexible multifunctional pan piezoelectric fiber with hydrophobicity, energy storage, and fluorescence. Polymers.

[21] Kesavan A.V., Kumar M.P., Rao A.D., Ramamurthy P.C. (2019). Light management through up-conversion and scattering mechanism of rare earth nanoparticle in polymer photovoltaics. Opt. Mater..

[22] Wang D.M., Yu Y.L., Ai X., Pan H.W., Zhang H.L., Dong L.S. (2019). Polylactide/poly(butylene adipate-co-terephthalate)/rare earth complexes as biodegradable light conversion agricultural films. Polym. Adv. Technol..

[23] Jadhav A., Pawar A., Hwang T.R., Lee J.W., Choi M.W., Kim B.K., Kang Y.S. (2012). Wavelength conversion using rare earth doped oxides in polyolefin based nanocomposite films. Polym. Int..

[24] Kamimura M., Kanayama N., Tokuzen K., Soga K., Nagasaki Y. (2011). Near-infrared (1550 nm) in vivo bioimaging based on rare-earth doped ceramic nanophosphors modified with PEG-b-poly(4-vinylbenzylphosphonate). Nanoscale.

[25] Li J., Wang J., Yu Y., Zhu Y.N., Ge M.Q. (2017). Preparation and luminescence properties of rare-earth doped fiber with spectral blue-shift: SrAl_2_O_4_:Eu^2+^, Dy^3+^ phosphors/triarylsulfonium hexafluoroantimonate based on polypropylene substrate. J. Rare Earths.

[26] Zhang Y.Y., Shen L.F., Pun E.Y.B., Chen B.J., Lin H. (2014). Multi-color fluorescence in rare earth acetylacetonate hydrate doped poly methyl methacrylate. Opt. Commun..

[27] Rao K.S.V.K., Liu H.G., Lee Y.I. (2010). Fluorescence spectroscopy of polymer systems doped with rare-earth metal ions and their complexes. Appl. Spectrosc. Rev..

[28] Wu L.Y., Chen G.M., Li Z.B. (2017). Layered rare-earth hydroxide/polyacrylamide nanocomposite hydrogels with highly tunable photoluminescence. Small.

[29] Zhao H., Hu J., Zhang Q.J., Bao J., Liu W.H., Gao C., Luo Y.H. (2006). Local microstructure characterization of rare earth-doped PMMA with low-ion content by fluorescence EXAFS. J. Appl. Polym. Sci..

[30] Gao Z.H., Wu Y.W., Xu L., Hao H.X., Wu Q.Y., Xie H.D. (2018). Preparation and luminescent properties of Eu(III) organic complex and novel transparent ethylene-methyl acrylate copolymer (EMA) films doped with complexes. Opt. Mater..

[31] Yu L.P., Zhang X., Wei D.X., Wu B., Jiang X.R., Chen G.Q. (2019). Highly efficient fluorescent material based on rare-earth-modified polyhydroxyalkanoates. Biomacromolecules.

[32] Zhang D.J., Zhao W.J., Feng Z.X., Wu Y.Z., Huo C.X., He L., Lu W.J. (2019). Preparation of polymer-rare earth complexes based on Schiff-base-containing salicylic aldehyde groups attached to the polymer and their fluorescence emission properties. e-Polymers.

[33] Wu Y.W., Hao H.X., Wu Q.Y., Gao Z.H., Xie H.D. (2018). Preparation and luminescent properties of the novel polymer-rare earth complexes composed of poly(ethylene-co-acrylic acid) and europium ions. Opt. Mater..

[34] Gao B.J., Zhang L.Q., Zhang D.D. (2018). Synthesis and characterization of two novel Schiff base type macromolecular ligands and preliminary research on luminescent property of polymer-rare earth complexes. J. Polym. Res..

[35] Zhang Y.X., Chen S.Y. (2018). Rare earth complexes using azobenzene-containing poly(aryl ether)s with different absorption wavelengths as macromolecular ligands: Synthesis, characterization, fluorescence properties and fabrication of fluorescent holographic micropatterns. RSC Adv..

[36] Ge J.C., Choi N.J. (2017). Fabrication of functional polyurethane/rare earth nanocomposite membranes by electrospinning and its VOCs absorption capacity from air. Nanomaterials.

[37] Gao B., Fang L., Men J. (2012). Studies on preparation, structure and fluorescence emission of polymer-rare earth complexes composed of aryl carboxylic acid-functionalized polystyrene and Tb(III) ion. Polymer.

[38] Gao B., Zhang L., Li Y. (2016). Effect of substituent groups with two types on benzene ring on photoluminescence property of complexes of benzoic acid—Functionalized polystyrene with Eu(III) ion. J. Photochem. Photobiol. A Chem..

[39] Yang F., Yuan Y., Sijbesma R.P., Chen Y.L. (2020). Sensitized mechanoluminescence design toward mechanically induced intense red emission from transparent polymer films. Macromolecules.

[40] Chinya I., Sen R., Dhar A. (2017). Synthesis and characterization of transparent erbium-ytterbium co-doped polymer nanocomposites for fabrication of polymer optical preform. Phys. Status Solidi A.

[41] El-Newehy M.H., Kim H.Y., Khattab T.A., El-Naggar M.E. (2022). Production of photoluminescent transparent poly(methyl methacrylate) for smart windows. Luminescence.

[42] Wang H.H., Yuen U.E. (2006). Synthesis of thermoplastic polyurethane and its physical and shape memory properties. J. Appl. Polym. Sci..

[43] Wang L.D., Tang J., Li R.Z., Zhang T., Tong L., Tang J., Xu L. (2017). Synthesis and characterization of electro-optic polyurethane-imide and fabrication of optical waveguide device. High Perform. Polym..

[44] Jang J.Y., Do J.Y. (2014). Synthesis and evaluation of thermoplastic polyurethanes as thermo-optic waveguide materials. Polym. J..

[45] Jiang Y., Da Z.L., Qiu F.X., Yang D.Y., Guan Y.J., Cao G.R. (2018). Azo biphenyl polyurethane: Preparation, characterization and application for optical waveguide switch. Opt. Mater..

[46] Kim M.S., Song M.Y., Jeon B., Lee J.Y. (2012). Synthesis and electro-optic properties of novel polyurethane containing the nitrophenylazoresorcinoxy group. Polym. Int..

[47] Qiu F.X., Zhang W., Liu J.H., Yang D.Y. (2010). Optically active polyurethane containing asymmetric center: Preparation, characterization and thermo-optic properties. Polym.-Plast. Technol. Eng..

[48] Moreno J., Arregui F.J., Matias I.R. (2005). Fiber optic ammonia sensing employing novel thermoplastic polyurethane membranes. Sens. Actuators B Chem..

[49] Robert V., Lemercier G. (2006). A combined experimental and theoretical study of carboxylate coordination modes: A structural probe. J. Am. Chem. Soc..

[50] Tsaryuk V.I., Zhuravlev K.P. (2021). Peculiarities of the luminescence excitation of europium and terbium substituted benzoates. J. Lumin..

[51] Binnemans K. (2015). Interpretation of europium(III) spectra. Coord. Chem. Rev..

[52] Azab H.A., Kamel R.M. (2016). Sensitive and selective fluorescent chemosensor for the detection of some organophosphorus pesticides using luminescent Eu(III) complex. J. Photochem. Photobiol. A.

